# High Prevalence of Pre-Existing Liver Abnormalities Identified Via Autopsies in COVID-19: Identification of a New Silent Risk Factor?

**DOI:** 10.3390/diagnostics11091703

**Published:** 2021-09-17

**Authors:** Yuri Hirayama, Natasha Faye Daniels, Shelley Evans, David Clarke, Stephenie Purvis, Charlotte Oliver, Stephen Woodmansey, Joy Staniforth, Elizabeth J. Soilleux

**Affiliations:** 1 School of Clinical Medicine, University of Cambridge, Cambridge CB2 0SP, UK; Yuri.hirayama@addenbrookes.nhs.uk (Y.H.); natasha.daniels3@nhs.net (N.F.D.); 2 Department of Pathology, University of Cambridge, Cambridge CB2 1QP, UK; sce30@cam.ac.uk (S.E.); stephenwoodmansey@hotmail.co.uk (S.W.); 3 Haematopathology and Oncology Diagnostic Service, Cambridge University Hospitals NHS Foundation Trust, Cambridge CB2 0QQ, UK; david.clarke@addenbrookes.nhs.uk (D.C.); Stephenie.purvis@addenbrookes.nhs.uk (S.P.); Joy.Staniforth@addenbrookes.nhs.uk (J.S.); 4 Department of Histopathology, Cambridge University Hospitals NHS Foundation Trust, Cambridge CB2 0QQ, UK; Charlotte.Oliver@addenbrookes.nhs.uk

**Keywords:** COVID-19, SARS-CoV-2, post-mortem, hepatic pathology, interferon dysregulation, liver function tests, steatosis, cirrhosis, autopsy, pathology

## Abstract

A high prevalence of hepatic pathology (in 17 of 19 cases) was reported in post-mortem (PM) examinations of COVID-19 patients, undertaken between March 2020 and February 2021 by a single autopsy pathologist in two English Coronial jurisdictions. The patients in our cohort demonstrated high levels of recognised COVID-19 risk factors, including hypertension (8/16, 50%), type 2 diabetes mellitus (8/16, 50%) and evidence of arteriopathy 6/16 (38%). Hepatic abnormalities included steatosis (12/19; 63%), moderate to severe venous congestion (5/19; 26%) and cirrhosis (4/19; 21%). A subsequent literature review indicated a significantly increased prevalence of steatosis (49%), venous congestion (34%) and cirrhosis (9.3%) in COVID-19 PM cases, compared with a pre-pandemic PM cohort (33%, 16%, and 2.6%, respectively), likely reflecting an increased mortality risk in SARS-CoV-2 infection for patients with pre-existing liver disease. To corroborate this observation, we retrospectively analysed the admission liver function test (LFT) results of 276 consecutive, anonymised COVID-19 hospital patients in our centre, for whom outcome data were available. Of these patients, 236 (85.5%) had significantly reduced albumin levels at the time of admission to hospital, which was likely indicative of pre-existing chronic liver or renal disease. There was a strong correlation between patient outcome (length of hospital admission or death) and abnormal albumin at the time of hospital admission (*p* = 0.000012). We discuss potential mechanisms by which our observations of hepatic dysfunction are linked to a risk of COVID-19 mortality, speculating on the importance of recently identified anti-interferon antibodies.

## 1. Introduction

Since its discovery in Wuhan in December 2019, the novel coronavirus SARS-CoV-2 has caused a global pandemic [1,2], resulting in over 183 million worldwide cases and over 3.9 million fatalities [3]. Most cases are mild and self-limiting, with symptoms such as pyrexia, cough, anosmia, and myalgia [4,5]. Risk factors for more severe disease have been identified, including increasing age, obesity, type 2 diabetes, hypertension, cardiovascular disease and chronic respiratory disease [6,7,8]. However, the interplay between these factors, potential causal relationships, and their roles in infection, disease progression and mortality, is not fully understood.

Careful analysis of past cases of severe COVID-19 can inform the global collaborative effort to understand precisely why severe disease develops in a minority of patients. Post-mortem (PM) studies are highly relevant to this process. The SARS-COV-2 virus gains access to host cells via the ubiquitously expressed ACE-2 receptor [9,10,11], and thereby has the potential to invade and affect any organ system. Moreover, severe cases are known to result in systemic immune and coagulation dysregulation, with reported consequences including acute respiratory distress syndrome (ARDS) [12,13] thrombosis [14], and damage to the cardiovascular, renal and hepatobiliary systems [15]. PM studies enable examination of these broad consequences of COVID-19, as well as allowing pre-existing, but potentially undetected, pre-/comorbidities to be described. A better understanding of pre-/comorbid risk factors and the mechanisms by which they contribute to severe disease could enable more effective treatment, as well as risk mitigation, providing a more robust evidence base for targeted public health measures, such as the shielding of particular groups. 

This report summarises the findings of 22 PM examinations carried out in two English Coronial jurisdictions between March 2020 and February 2021. The deceased individuals had either an antemortem confirmation of COVID-19 or a compatible history, together with PM histopathological changes in the lung consistent with COVID-19. We note a high frequency of likely pre-existing liver disease, which is hitherto largely undescribed, and question whether this might represent an asymptomatic premorbid risk factor for severe COVID-19. 

## 2. Methods

Twenty-two clinical autopsies of cases with confirmed or likely COVID-19, undertaken by an experienced consultant autopsy pathologist (E.J.S.) and carried out in two English Coronial jurisdictions between March 2020 and February 2021, were analysed and the findings summarised. The liver was only examined in 19/22 cases. As this was an anonymised audit project, neither specific ethical approval nor consent from next-of-kin were required.

**Figure 1:**
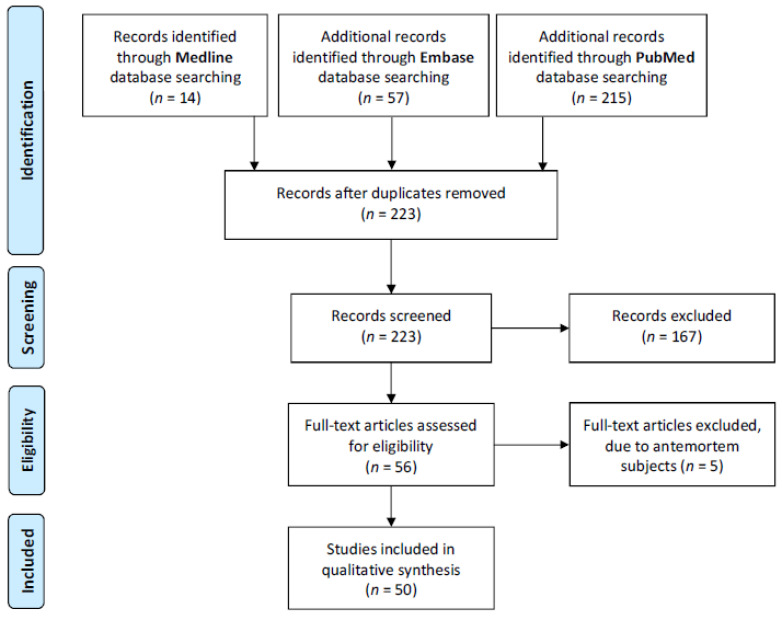
PRISMA flow diagram indicating strategies for literature search [16].

To place these results in the context of the global COVID-19 pandemic, a literature review was conducted on PubMed, Embase and Ovid MEDLINE, in accordance with the Preferred Reporting Items for Systematic Reviews and Meta-Analyses (PRISMA) guidelines [16], in order to identify similar reports of PM examinations in COVID-19. All English-language articles published before 3 August 2020 were eligible for inclusion and antemortem studies were excluded. The search strategy was to search for the following set of terms: 1. Coronavirus OR (corona virus) OR COVID-19 OR SARS-CoV-2; 2. Postmortem/ OR autopsy/; 3. 1 AND 2. Two reviewers (Y.H. and J.S.) screened the titles and abstracts of the search results. Disagreements over the inclusion of papers were discussed by all authors until mutual agreement was reached (Figure 1). The macroscopic and microscopic findings for each organ system were extracted and summarised.

To investigate liver pathology, the number of post-mortem studies identified by a literature search that specifically mentioned liver findings was recorded and a more detailed summary of these hepatic findings was produced. Next, we sought to investigate the hypothesis that hepatic pathology may be more prevalent among individuals who go on to die as result of COVID-19 than among those who die from other causes. Pre-pandemic post-mortem studies were identified and data on the frequency of key hepatic pathologies seen at autopsy (steatosis, cirrhosis, and venous congestion) were extracted. The total frequency of these hepatic pre-pandemic post-mortem abnormalities was calculated and compared to their frequency in COVID-19 post-mortem cases, including our own, reported here. Statistical analysis was performed using the Chi-squared test on Microsoft Excel.

Under ethical approval IRAS 162057, retrospective analysis of admission liver function test (LFT) results, specifically alanine transaminase and albumin, was undertaken for 276 consecutive, anonymised COVID-19 positive inpatients in Addenbrooke’s Hospital, Cambridge, between 1 March 2020 and 3 June 2020, for whom outcome data were known. All patients had a positive Eurofins PCR-based test for SARS-CoV-2 within the 10 days preceding admission or on the date of admission. Abnormal LFT results were defined as ALT > 40 iμ/L and albumin < 35 g/L. The length of hospital stay in days and mortality were also recorded. A Chi-squared test was used to compare the frequency of abnormal results between patient groups, which were stratified by stay length or mortality. A one-way ANOVA was performed to determine the correlation between admission LFT data and stay length or mortality. A *p*-value of <0.05 was considered statistically significant for all tests. 

## 3. Results

**Figure 2:**
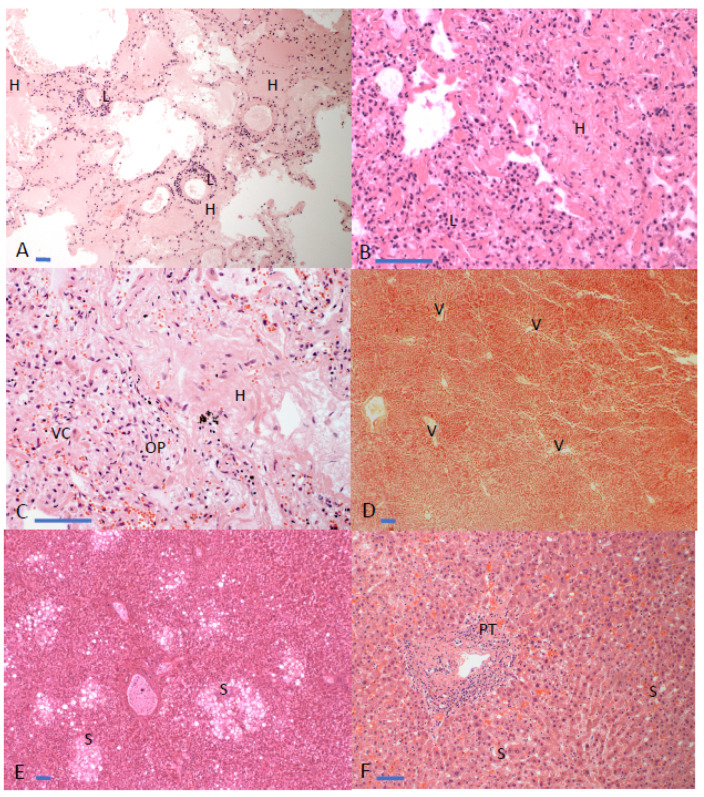
Examples of pulmonary and hepatic pathology from COVID-19 post-mortem cases described in Table 1. All images are of hematoxylin and eosin (H&E)-stained formalin-fixed, paraffin-embedded tissue sections. (**A**) Lung (10× objective) showing acute respiratory distress syndrome (ARDS)/diffuse alveolar damage (DAD), with abundant hyaline material (H), forming hyaline membranes within alveoli and/or completely filling them. Moderate numbers of lymphocytes (L) are seen. (**B**) Higher magnification (40× objective) image of lung from a different autopsy case showing similar features, such as hyaline material (H), but with more admixture of macrophage/histiocytic cells where lymphocytes (L) are marked. (**C**) Lung (40× objective) showing hyaline material, but also an area of early organizing pneumonia (OP), as well as desquamated pneumocytes and/or macrophages with viral cytopathic change (VC). (**D**) Liver (10× objective) showing passive venous congestion with distended sinusoids, giving an overall lobular pattern, and dilated central veins (V). (**E**) Liver (10× objective) with marked steatosis (S) or “fatty change”. (**F**) Liver (20× objective) with more subtle steatosis (S) and some portal tract (PT) expansion by fibrous tissue, as is seen in the early stages of progression of steatosis to cirrhosis.

The findings of the 22 PM cases (14 male; 8 female) are detailed in Table 1. Notable features on external examination were cyanotic fingernails (15/19; 79% (3 cases could not be assessed due to racial pigmentation)) and a hyperinflated chest (6/22; 27%). The diagnosis of COVID-19 was made either by antemortem or PM viral PCR, or on the basis of characteristic PM histopathological findings, including diffuse alveolar damage/acute respiratory distress syndrome spectrum changes and lymphohistiocytic infiltration with viral cytopathic effects, but generally few neutrophils (Figure 2A–C). The age range in years was 48–94, with a mean age of 70. The primary cause of death stated on the UK death certificate, as given by the pathologist, was of a respiratory nature in 19 cases (‘ARDS, viral pneumonia’= 3; ‘acute bacterial pneumonia’ = 3; ‘Viral pneumonia with features consistent with COVID-19′ = 2; ‘COVID-19 infection’ = 7; ‘pulmonary thromboembolism’ = 4). The primary cause of death in two cases was cardiac arrhythmia, and in one case it was hypoxic brain injury and multiorgan failure. COVID-19 infection, either probable or confirmed, was specifically mentioned in the causes of death in 19 of the 22 cases, under part 1a in 11 cases, part 1b in 3, part 1c in 4 and part 2 in 1. The three cases in which COVID-19 infection is not mentioned on the death certificate were undertaken in March 2020, at the start of the pandemic in the UK, when COVID-19 testing was not widely available and pathologists were less familiar with COVID-19 histopathological findings in the lung and, thus, a description of the lung findings (either ARDS or organising pneumonia) was included on the death certificate. Re-review of these cases in view of subsequent detailed publications describing COVID-19 lung pathology [17,18,19,20,21,22,23,24,25,26,27,28,29,30,31,32,33,34,35,36,37,38,39,40,41,42,43,44,45,46,47,48,49,50,51] indicated that the cause of death was COVID-19.

As these were coronial (medicolegal) autopsies, the quantity and quality of clinical information available, such as past medical history, depended on the deceased’s recorded interactions with health services. For the 16 cases where the past medical history was available, 16 cases had pre-existing comorbidities known to increase the risk of severe COVID-19 [6,7,8,53], including diabetes (*n* = 8), hypertension (*n* = 8), cardiovascular disease (*n* = 6), chronic kidney disease (*n* = 4) and interstitial lung disease (*n* = 1). Likewise, established potentially fatal complications of SARS-CoV-2 infection were observed microscopically, including ARDS (*n* = 17/21 cases undergoing histological examination), thromboembolism (*n* = 8/21) and consequent bacterial pneumonia, described either as the presence of bronchopneumonia or neutrophils, superimposed on the ARDS picture (*n* = 9/21) [12,13,14,54] (Figure 2A–C). The most pertinent cardiovascular findings were cardiomegaly and ventricular dilatation, which is in keeping with hypertension and cardiovascular disease being key risk factors for severe disease. Of the 22 patients, 15 (68%) had cardiomegaly, which we chose to define as >90th centile for gender and body weight [52], with 12/22 (55%) cases above the 97th centile. Twenty (91%) patients had moderate or severe ventricular dilation. 

Notably, no patients had a formal antemortem diagnosis of liver disease, yet hepatic abnormalities were detected at PM in 17/19 (89%) of the cases in which the liver was examined (Figure 2D–F; Table 1). The liver was not examined in three cases because, early in the pandemic, limited autopsy examinations were undertaken due to fears about risks to pathologists and anatomical pathology technicians. Only the heart and lungs were examined in these three cases. Thirteen out of nineteen cases (68%) demonstrated likely longstanding liver pathology in the form of steatosis (nine cases without cirrhosis; three cases with cirrhosis) and cirrhosis without obvious steatosis (one case.) Additional hepatic abnormalities detected included four cases showing passive venous congestion without obvious evidence of either steatosis or cirrhosis, two cases showing passive venous congestion with steatosis, while one individual had steatosis, cirrhosis and passive venous congestion. In five of these seven cases with passive venous congestion, there was autopsy evidence of significant ischaemic heart disease, with one case additionally having interstitial lung disease, indicating that the passive venous congestion was likely to be longstanding. In one case without any macroscopically obvious liver pathology, a microscopically identified portal tract lymphocytic infiltrate was present and, given the patient’s history of intravenous drug use, hepatitis C was clearly a possibility, but, by the time the histological material was prepared for the pathologist’s review, no suitable sample for hepatitis testing remained. In this case, the liver also appeared pale and acutely ischaemic macroscopically as a consequence of shock, due to a large, multifocal gastrointestinal haemorrhage, which was assumed to be a consequence of enterocyte infection with SARS-CoV-2.

Table 2 [17,18,19,20,21,22,23,24,25,26,27,28,29,30,31,32,33,34,35,36,37,38,39,40,41,42,43,44,45,46,47,48,49,50,51,55,56,57,58,59,60,61,62,63,64,65,66,67,68,69] summarises the key macroscopic and microscopic findings derived from published PM studies of COVID-19 and indicates that our findings were in keeping with those of other autopsy studies. Amongst these studies, there was considerable heterogeneity in terms of pathological findings, most notably the presence or absence of myocarditis, vascular inflammation and /or microthrombi. A total of 42% of the studies specifically examined the hepatic system, giving heterogeneous results, with variable frequencies of cirrhosis/ fibrosis, steatosis, venous congestion, inflammatory changes and hepatocyte injury or death (Table 3) [17,18,19,20,21,22,23,24,25,26,27,28,29,30,31,32,33,34,35,36,37,38,39,40,41,42,43,44,45,46,47,48,49,50,51,55,56,57,58,59,60,61,62,63,64,65,66,67,68,69].

The prevalence of steatosis, venous congestion and cirrhosis was compared between published COVID-19 PM cohorts (Table 3), our PM cohort (Table 1) and pre-pandemic PM cohorts (Table 4) [17,18,19,20,21,22,23,24,25,26,27,28,29,30,31,32,33,34,35,36,37,38,39,40,41,42,43,44,45,46,47,48,49,50,51,55,56,57,58,59,60,61,62,63,64,65,66,67,68,69,70,71,72,73,74,75,76]. There was a very significant increase in steatosis and cirrhosis among patients dying of COVID-19 in our study and in published COVID-19 autopsy studies, compared with pre-pandemic PM cohorts. While there was no significant increase in the frequency of venous congestion between our COVID-19 patients and pre-pandemic PM cohorts, there was a statistically significant difference between the frequency of venous congestion in published COVID-19 autopsy studies and the frequency in pre-pandemic PM cohorts.

To corroborate our observation of increased levels of chronic hepatic pathology in COVID-19 patients, we decided to interrogate patient LFT results for all consecutive COVID-19-positive inpatients, for whom we could obtain admission LFT data (alanine transaminase (ALT) and albumin, alkaline phosphatase (ALP)), length of hospital stay and survival data (*n* = 276; average age 71 years; 109 (39%) female; 167 (61%) male). Of the admitted patients, 88 (32%) died in hospital and, of these, 62 (70%) were male; however, there was no overlap between patients dying in this cohort and the autopsy cohort described above. Only admission LFT blood sample data was used as we reasoned that, at later time points, there would be a greater risk that LFT abnormalities might be confounded by SARS-CoV-2 infection rather than indicating any underlying chronic pathology. Of the 276 patients, 258 (93%) had at least one abnormal LFT parameter. We used albumin (half-life: 20 days) as a marker of long-term liver (or renal) disease and ALT (half-life: 47 h) as an indicator of more recent changes to hepatic function [77]. We compared the albumin and ALT levels between the patient groups with four different lengths of stay in hospital (<7 days, 7–13 days, 14–21 days and >21 days), demonstrating a statistically significant decrease in the albumin levels, with poorer outcomes (i.e., increasing lengths of hospital stay and death) (*p* = 0.000012) (Table 5). However, no statistically significant difference was found in the ALT levels between these groups *(p =* 0.73), indicating that the admission albumin level was a predictor of COVID-19 severity. However, higher ALT levels showed a stronger correlation with those patients who went on to die in hospital, compared with those who survived (*p =* 0.0135), than did lower albumin levels (*p =* 0.081). This corroborates the fact that ALT levels are likely to act as a marker of acute pathology related to SARS-Co-V2 infection, while albumin is more likely to indicate pre-existing pathological conditions that act as risk factors for more severe SARS-Co-V2 infection.

## 4. Discussion

Herein we report the findings of 22 PM examinations carried out on confirmed or suspected COVID-19 cases. Of the 19 cases in which the liver was examined, there was a striking over-representation of hepatic pathology, with at least one likely longstanding liver abnormality in each case. There were several limitations to this observational study, including the small sample size, its limitation to those whose autopsies were requested by a coroner and its limitation to two Coronial jurisdictions, which may mean that this cohort is not necessarily representative of all deaths due to COVID-19 in the U.K. Moreover, the autopsies were carried out by one pathologist, which increased the risk of reporting bias. Furthermore, a subsequent literature review identified the widespread reported multisystemic effects of COVID-19 documented thus far in PM studies. We used pre-COVID-19 pandemic PM studies with a range of causes of death to gain an indication of the baseline level of hepatic pathology amongst the global population. Although our findings were restricted by the limitations of literature reviews, such as the quality of evidence and biases such as selection and publication bias, our results indicate a higher prevalence of hepatic pathology in individuals dying from COVID-19 than from other causes. On the basis of the observational data reported, no causal inference can be made as there were potential confounding factors in our comparison with pre-pandemic autopsy data, such as age, ethnicity and geographical and socioeconomic background. However, our findings indicate that liver disease may be a more important risk factor for COVID-19 mortality than previously thought, which should encourage further studies to explore this possible link with a larger and globally representative sample size.

To corroborate our findings in autopsy data indicating that chronic liver disease is a significant risk factor for mortality in COVID-19, we analysed admission LFT data. Admission albumin levels, a crude and incompletely specific indicator of chronic liver disease, predicted more severe SARS-Co-V2 infection, with longer hospital admission. This was in agreement with previous studies [78,79,80], including those confirming hypoalbuminaemia as an independent predictive factor for mortality in COVID-19 patients [79]. As a comparator, we analysed ALT levels, which indicated much more acute hepatic pathology, and found that admission ALT did not predict the length of hospital admission. However, commensurate with their ability to indicate acute pathology, raised admission ALT levels correlated with the risk of mortality, presumably acting as a biomarker of widespread direct, or indirect, COVID-19-mediated injury to the organs, including the liver. 

Emerging evidence supports the hypothesis that liver disease of all stages, ranging from steatosis to cirrhosis, predisposes affected patients to severe SARS-CoV-2 infection. Non-alcoholic fatty liver disease (NAFLD), also known as metabolic-associated fatty liver disease (MAFLD), is thought to be the most common cause of liver disease in the western world, causing a spectrum of hepatic dysfunction ranging from to steatosis to cirrhosis [81]. A recent systematic review identified MAFLD as being associated with a four-to-sixfold increase in the risk of severe COVID-19 [82] and a further study reports that MAFLD independently increases the risk of severe COVID-19, after adjusting for the effect of sex, obesity and diabetes, with the risk increasing in line with the degree of resultant liver fibrosis [83]. Univariate analysis of UK Biobank data confirms that liver fat content correlates with the risk of symptomatic COVID-19 [84]. While it might be argued that liver fat content is simply a surrogate marker for overweight individuals, the same study showed that overweight individuals were only at increased risk of more severe COVID-19 if they demonstrated concurrent raised hepatic fat levels [84], suggesting liver dysfunction may form part of the mechanism by which obesity confers COVID-19 risk.

Hepatic dysfunction due to ethanol consumption in alcoholic liver disease (ALD) also causes a spectrum of liver disease, ranging from steatosis to cirrhosis [85], and appears to predispose affected patients to severe COVID-19 [86]. Alcohol consumption disrupts both the innate and adaptive immune system and increases susceptibility to both viral and bacterial infections [87]. Moreover, like MAFLD, ALD often co-exists with other COVID-19 risk factors, such as metabolic syndrome; occasionally, ALD patients may be immunocompromised as a result of alcohol-related hepatitis treatment with corticosteroids [88].

The exact mechanisms by which liver dysfunction predisposes affected patients to severe COVID-19 are unknown but may be immunological. Cirrhosis dysregulates both innate and adaptive immunity, increasing susceptibility to acute inflammatory reactions and subsequent exaggerated courses [89]. It has been reported as an independent risk factor for mortality in patients with Acute Respiratory Distress Syndrome (ARDS) [90] and a predictor of adverse outcomes in Systemic Inflammatory Response Syndrome (SIRS) [91], both ARDS and SIRS being potential COVID-19 sequelae [12,92]. Importantly, multivariable adjusted models have been used to demonstrate that liver fat levels correlate with multiple markers of inflammation and oxidative stress, whereas obese patients with normal livers, who may not be at increased risk of severe COVID-19 [84], are characterised by lower pro-inflammatory cytokine levels [93].

One other possibility is that hepatic inflammation or dysfunction may play a role in hampering interferon (IFN)-mediated anti-viral responses [94,95]. Neutralising IgG auto-antibodies against type 1 IFNs have been found in individuals with severe COVID-19, but not in those with mild disease [96]. These antibodies prevented IFNs from blocking SARS-CoV-2 cell entry in vitro and reduced the levels of circulating IFN in vivo to low or undetectable levels. For example, when IFN-alpha was used in the treatment of viral hepatitis, its efficacy was diminished in alcohol-dependent patients; this was hypothesised to have been the result of ALD-mediated down-regulation of IFN-alpha signalling pathways, although anti-IFN antibodies have not been specifically sought in this setting [97]. Trials of IFN as a COVID-19 therapy have thus far yielded uncertain results [98]. The link between hepatic dysfunction and IFN dysregulation in the context of severe COVID-19 therefore requires further investigation, as this relationship has mechanistic, prognostic and therapeutic implications.

In summary, our study indicates that liver disease is a potentially important COVID-19 risk factor that may be undetected prior to autopsy. Whilst the global prevalence of ALD is difficult to estimate, steatosis is thought to develop in over 90% of heavy drinkers [99] and metanalysis estimates the global prevalence of MAFLD at approximately 25% [100]. However, since the early stages of both alcoholic and non-alcoholic fatty liver disease are usually asymptomatic [88], neither disease is as widely recognised or as frequently investigated as other commonly cited COVID-19 risk factors, such as age, obesity and hypertension. Given its high prevalence, its potential role in mediating and/or predisposing affected patients to severe COVID-19 and its potentially asymptomatic [88] and reversible [101] nature in the early stages, greater emphasis should be placed on screening for liver disease when considering public health measures, such as shielding and risk stratification.

## Figures and Tables

**Table 1:** Key findings of 22 post-mortem examinations carried out on confirmed or suspected cases of COVID-19 infection between March 2020 and February 2021. Centiles for heart weights were derived in light of biological sex and body weight [52].

		Case 1	Case 2	Case 3	Case 4	Case 5	Case 6	Case 7	Case 8	Case 9	Case 10	Case 11	Case 12	Case 13	Case 14	Case 15	Case 16	Case 17	Case 18	Case 19	Case 20	Case 21	Case 22	Key Findings Summary for Cases 1–22
**Age, race biological sex**		57y Cn M	79y Cn M	48y Cn F	82y A-C/ mixed race M	57y A-C M	89y Cn M	81y A-C M	72y Cn M	92y Cn M	48y Cn M	76y Cn M	42y A-C F	83y A-C M	86y Cn F	85y Cn F	67y Cn F	57y A-C F	94y Cn F	56y Cn M	65y Cn M	69y Cn F	59y Cn M	14/22 (64%) M; mean age 70y (SD: 16); 16/22 (73%) Cn
**Diagnosis of COVID-19**		History & histol	History & histol	AM RNA test	History & histol	History & histol	AM RNA test	AM RNA test	AM RNA test	AM RNA test	History & histol	History & histol	PM lung RNA test	AM RNA test	AM RNA test	PM RNA test	Histol	Histol	Histol	PM RNA test	Histol	AM RNA test	PM lung RNA test	
**MCCD section I (a): Disease or condition leading directly to death**		ARDS	ARDS	Cardiac arryth-mia	PE & viral Pu, consis-tent with COVID-19	PE	Acute bacterial Pu	Viral Pu with features consis-tent with COVID-19	COVID-19 infection	COVID 19	GI haemo-rrhage, ARDS, excess-ive blood levels of meth-adone	RHF leading to cardiac arrhyth-mia	PE	PTE & COVID-19	COVID-19 & bacterial BPu	COVID-19 & IHD	Hypoxic brain injury, multi-organ failure	COVID-19	Acute bacter-ial Pu	Multi-ple bil-ateral PTE	COVID-19	COVID-19	COVID-19 pneu-monitis	6/22 (27%) thrombo-embolic complica-tion of COVID-19 included in MCCD Part I
**MCCD section I (b): Disease/** **condition leading to I (a)**		Pu	Pu	RHF	DVT	DVT	COVID-19 pneumo-nitis and aspirat-ion of food material	*n/a*	*n/a*	*n/a*	COVID-19	Pulm-onary artery thromb-osis	DVT	DVT	*n/a*	*n/a*	Cardiac arrest due to arrhyth-mia	*n/a*	COVID-19	DVT	*n/a*	*n/a*	*n/a*	
**MCCD section I (c): Disease/** **condition leading to I (b)**		*n/a*	*n/a*	OPu, mild lympho-cytic myo-carditis	*n/a*	Mild lympho-cytic Pu, consis-tent with resolving COVID-19	*n/a*	*n/a*	*n/a*	*n/a*	*n/a*	ARDS/ viral pneu-monitis, consis-tent with COVID-19	COVID-19	*n/a*	*n/a*	*n/a*	*n/a*	*n/a*	*n/a*	COVID-19	*n/a*	*n/a*	*n/a*	
**MCCD section II: Contribut-ing to but not directly causative of death**		*n/a*	*n/a*	T2DM, hypo-thyroid-ism and Rett’s syn-drome	DM, demen-tia, frailty	*n/a*	IHD, Parkin-son’s disease	CHF, IHD, T2DM, CKD	IHD, T2DM, CKD	ILD, COPD, iron deficien-cy anaemia, IHD, aortic valve disease	*n/a*	*n/a*	*n/a*	CKD	CHF, AF, IHD, COPD	*n/a*	Bilateral BPu, arising on a back-ground of COVID-19	*n/a*	*n/a*	*n/a*	*n/a*	*n/a*	HTN	
**Past medical history in addition to MCCD**		HTN, high chol-esterol, prostat-ectomy, gout	IgG MGUS, AF, HTN, border-line DM, CKD, spinal degen-eration, neuro-pathy	Nil addit-ional	HTN, T2DM	*not known*	AF, CABG ×3	End stage CKD, vascular demen-tia, stroke, HTN, epilepsy, blind-ness, cataracts, divertic-ular disease, gout	High chol-esterol, duo-denal ulcera-tion, IBS, MI, CABG ×4, PF	Demen-tia	*not known*	Uro-sepsis, AKI, Alz-heimer’s & vascular demen-tia, falls, T2DM	HTN	T2DM, HTN, Stage 3 CKD	Re-current UTIs, osteo-porosis	Drug eluting CA stent, hip replace-ment	Asthma, scoliosis, osteo-arthritis, cholecyst-ectomy	*not known*	*not known*	*not known*	Obesity, HTN, T2DM	CKD, hyper-thyroid-ism, HTN, arthritis, gout, major depress-ion	*not known*	8/16 (50%) HTN; 8/16 (50%) DM; 6/16 (38%) arterio-path
**BMI (kg/m^2^)**		27.2	31.3	24.8	16.8	29.0	19.2	19.4	30.3	19.4	21.0	18.4	32.5	24.4	23.2	19.2	25.2	38.2	21.4	26.7	27.5	20.0	35.6	Mean 25, S.D. 5.9
**Cardiovascular**	Heart weight/g (cent-ile)	400 (50–90)	610 (>97)	320 (50–90)	355 (>97)	460 (>97)	450 (>97)	350 (>97)	799 (>97)	508 (>97)	330 (90–97)	380 (90–97)	425 (>97)	500 (>97)	270 (10–50)	460 (>97)	320 (50–90)	455 (50–90)	280 (10–50)	510 (>97)	354 (10–50)	425 (90–97)	845 (>97)	12/22 (55%) >97; 3/22 (14%) 90–97; 4/22 (18%) 50- 90
	CA sten-osis	+	*N.S.*	*N.S.*	+	+	+++	+++	+++	++	+	+	*N.S.*	++	++	++	++	+	++	++	+	*N.S.*	+	3/22 (14%) +++; 9/22 (41%) ++ to +++
	Ventri-cles	Dilated L+/R++, sub-endo-cardial pallor	LVH	Dilated L++/ R+++, pallor, MNI+	Dilated L++/ R+++, pallor	Dilated L+, pallor, MNI+	Dilated L+++/ R+++, MH+++, MF++	Dilated L+++/ R+++, MH++, MF++	Dilated L+++/ R+++, MH+, MF+++, peri-cardial fibrosis +++	Dilated L++/R++, sub-endo-cardial pallor, MF++	Dilated L+++/ R+++; MNI+++	Dilated L+++/ R+++, MF+	Dilated L++/R++, pallor	LVH, dilated L+/R++	Dilated L++/R++	Dilated L+++/ R+++, pallor, MF+	Dilated L++/R+++; MF+	Border-line LVH	Dilated L+/R+++	LVH, dilated R+++	Dilated L++/R++, MF+, scattered neutro-phil infiltrate	Dilated L+/R+++, pallor	LHV, dilated L+++, MH+, MF++	20/22 (91%) >++ dilation. 9/22 (41%) IHD; 8/22 (36%) pallor
	Aortic athero-scler-osis	*N.S.*	*N.S.*	*N.S.*	+	+	+++	+++	++	*N.S.*	*N.S.*	+	*N.S.*	++	++	++	++	+	+	+	++	+	+++	9/22 (41%) ++ to +++
**Respiratory**	Key macro-scopic	ARDS	ARDS	ARDS, PO	ARDS, PO, PEs	ARDS, haemorr-hage, pus, PEs	ARDS, pus	ARDS, PO	ARDS, Pl adhes-ions	ARDS, emphy-sema, Pl eff, PEs	ARDS, emphy-sema, Pl eff, PEs	ARDS, Pl eff, PEs	PO, possible ARDS	Em-balmed, PEs, possible ARDS	Consoli-dation, possible ARDS, pus, PF, Pl adhes	ARDS	Consoli-dation, possible ARDS, pus, PO, Pl adhes, PEs	ARDS	ARDS, bronch-itis	ARDS, PO, PEs	ARDS, PO	ARDS, PO	ARDS, PO	22/22 (100%) ARDS (macro &/ micro); 8/22 (36%) PE
	Key micro-scopic	ARDS +++	ARDS +	OPu+++	ARDS +, OPu+++	OPu+	OPu+, arterial recanalis-ation+, pHTN+	ARDS +++	ARDS +++, arterial recanalis-ation+, pHTN+	OPu+, PF+, pHTN+	ARDS +++, BPu+	ARDS +, PF+, pHTN+, old infarcts+	ARDS +	ARDS +++, OPu+	ARDS +, OPu++	ARDS +, PF+, emphy-sema+	ARDS +, OPu+	ARDS +++, BPu+	ARDS +++, BPu+, emphy-sema+	*n/e*	ARDS +++, multi-nuclear macro-phages ++, thrombi, hyper-inflation	ARDS +, OPu+, hyper-inflation	ARDS +++, viral inclus-ions (type 2 pneum-ocytes)	
**Liver**		S(ma)+	C	VC+++	*n/e*	*n/e*	S(ma)+, VC+, C, PL0	VC+++	S(ma) +++, C	VC+++	Ischae-mic; PL0, paren-chymal L0	VC++, PL0	S(ma) +++	S(ma)+, C	Normal	S(ma)+	S (ma)++, ischaemic	S(ma) +++	S(ma)+, VC+	*n/e*	S(ma)+++	S(ma)+, VC+++	S(ma)+	12/19 (63%) S(ma); 5/19 (26%) VC++/ +++; 4/19 (21%) C

Key: + = mild, ++ = moderate, +++ = severe. For coronary artery stenosis, mild = <20%, moderate = >50% and severe = >70%. The term (mic) denotes microscopic findings. Abbreviations in table (alphabetical order): A-C = Afro-Caribbean; AF = atrial fibrillation; AKI = acute kidney injury; ARDS = acute respiratory distress syndrome; AM = antemortem; BMI = body mass index; BPu = bronchopneumonia; C = (hepatic) cirrhosis; CA = coronary artery; CABG = coronary artery bypass grafting; CHF = congestive heart failure; CKD = chronic kidney disease; Cn = Caucasian; COPD = chronic obstructive pulmonary disease; DM = diabetes mellitus; DVT = deep vein thrombosis; F = female; GI = gastrointestinal; Histol = histology; HTN = hypertension; IBS = irritable bowel syndrome; IgG MGUS = immunoglobulin G monoclonal gammopathy of undetermined significance; IHD = ischaemic heart disease; ILD = interstitial lung disease; L0 = lymphocytes; LVH = left ventricular hypertrophy; M = male; MCCD = Medical Certificate of Cause of Death; MF = myocardial fibrosis; MH = myocyte hypertrophy; MI = myocardial infarction; MNI = mononuclear (myocardial) infiltration; *n/a* = not applicable; *n/e* = not examined; *N.S.* = nil significant; OPu = organising pneumonia; PE = pulmonary embolism; PF = pulmonary fibrosis; pHTN = pulmonary hypertension; Pl adhes = pleural adhesions; Pl eff = pleura; effusion; PL0 = portal lymphocytic infiltrate; PM = postmortem; PO = pulmonary oedema; PTE = pulmonary thromboembolism; Pu = pneumonia; RHF = right-sided heart failure; RNA = ribonucleic acid; S(ma) = (hepatic) macrovesicular steatosis; S.D. = standard deviation; T2DM = type 2 diabetes mellitus; UTI = urinary tract infection; VC =venous (hepatic)congestion (passive in nature).

**Table 2:** Post-mortem pathology in COVID-19 described in published studies.

System	Site/Feature	Key Findings (Positive and Negative)
Cardiovascular	Pericardium	Effusion [17,19,55]; fibrinous pericarditis [23]
	Heart weight & chambers	Cardiomegaly/chamber hypertrophy [18,19,21,23,24,25,26,27,34] Chamber dilatation [17,18,19,20,21,22,26]
	Myocardial ischaemia	Infarction [24,27,31,56]; focal ischaemia, not otherwise specified [18] Scarring/ fibrosis [21,22,23,29,30,34,35]
	Cardiac inflammation	Lymphocytic myocarditis [23,25,28,29,33,35,55] Chronic inflammatory cells without myocarditis [18,23,26,36]
	Atherosclerosis	Significant coronary artery atherosclerosis [23] Atherosclerosis, not otherwise specified [22,24,27,28,29]
Vascular abnormalities (multiple systems)	Vasculitis	Endothelialitis of heart, small bowel, lung [55]; fibrinoid alteration (exact location not specified) [35]
	Microthrombi	Lung, glomeruli, spleen, heart, dermis, testis, liver sinusoids [35]
	Macroscopic thromboemboli	Deep venous [27]; pulmonary [18,21,29,38]
Respiratory	Pleura and pleural cavity	Effusions [17,19,21,23,25,32,41,43]; pleuritis [51]; thickening [17,19,21]; adhesions [23,30] Subpleural petechiae [36]
	Lung weights	Elevated [17,18,19,20,21,22,23,28,29,36,37,39,40,41,51,55]
	Lung cut surface	Red, consolidated, solidified [18,19,22,24,26,27,28,29,30,36,37,39,41,42,51,55,58] Haemorrhage [27,36]; oedema [23]; necrosis [27]; collapse [43] New fibrosis, extensive [23,38] Pre-existing structural lung disease [23,25,27]
	Airways	Upper airway inflammation [23,27,29,40,51,67] Bronchial inflammation [21,22,28,29,40,44,45,55] with mucus [19,26,30,40,46] Small airway acute inflammation (bronchopneumonia) [23,24,25,26,27,29,36,49] Aspiration pneumonia [40] No inflammation [17,32,37,40]
	Alveoli/interstitium	Diffuse alveolar damage/ARDS spectrum [17,18,19,20,21,22,23,24,26,27,28,29,30,31,32,33,34,35,36,37,38,39,40,41,42,43,44,45,46,47,48,49,50,51] Fibrosis [25,43,48,51] Lobar pneumonia [23] Chronic inflammatory cell infiltrate [17,18,19,22,23,24,27,28,29,30,33,34,39,41,42,47,49,56] Features suggestive of viral cytopathic effect [18,19,23,28,30,33,34,35,37,42,43,48,51]
	Vascular	Thrombosis with microangiopathy [18,19,20,21,22,24,27,28,31,35,37,39,45,46,47,49] Thrombosis [24,26,36,38,40,42,48,59] Small vessel fibrinoid necrosis [34] Severe endothelial injury with intracellular virus by EM [20,39] Neutrophilic & exudative capillaritis [18,20,21,22,29,32,33,45,46,47,48] Lymphocytic vasculitis [51] No vasculitis [49]; no thrombi [23]
Kidneys	Vascular	Pallor/ shock/ ischaemia [24,27,41,61,62]; infarction [24,27,62]; medullary congestion [41] Arteriolar & arterial fibrin thrombi [31,61] Microthrombi in glomeruli1 [7,22,24,31,35,61] Glomerular capillary congestion [51,61] Peritubular capillary congestion [51,61]
	Tubulo-interstitial	Acute tubular injury/ necrosis [21,24,28,35,42,44,51,60,61,62] ACE2 expression upregulated in proximal tubules [61] Acute tubulointerstitial nephritis61; no interstitial nephritis [62]
	Other	Nephrosclerosis [18,21,23,32,40,60] Other chronic kidney/ glomerular disease [24,35,40,43,60,61] Pyelonephritis [61]
Lymphoreticular	Spleen	Splenomegaly [18,20,23,27,37,50]; congestion [18,21,29,37,50]; diffluence [50] Red pulp infarction [50]; red pulp lymphoplasmacytic infiltrate [18] Atrophy [18,21,28,35,37,50]; white pulp hyperplasia [50] Haemophagocytosis [17,50] Acute splenitis (concurrent bronchopneumonia) [24]
	Lymph nodes & bone marrow	Hilar & mediastinal lymphadenopathy [21,24,50] & with haemophagocytosis [50] Lymph node haemophagocytosis (site not stated) [17]; no lymph node haematophagocytosis [50] Bone marrow haematophagocytosis [49]; no bone marrow haematophagocytosis [50]
Adrenal glands	Shocked appearance [25]; microscopic haemorrhage [37]; zona reticularis hyperplasia [21] Acute fibrinoid arteriolar necrosis [64] No adrenal abnormalities [50]	

**Table 3:** Post-mortem hepatic pathology in COVID-19 described in published studies.

Pathology			Ref & Frequency Where Ascertainable		Applied Interpretation
Fibrosis	Periportal		3/11 [21]	29/48 [59]	Pre-existing hepatic disease
	Incomplete septa		3/11 [21]	8/48 [59]	
	Cirrhosis		4/80 [29] 1/2 [32]	¼ [34]	
Steatosis	Centrilobular		1/1 [22]	½ [32]	Likely pre-existing alcoholic/non-alcoholic (e.g., obesity or diabetes-related) fatty liver disease
	Macrovesicular only		1/4 [34]	1/7 [33] 1/48 [59]	
	Microvesicular only		3/48 [60]		
	Mixed macrovesicular & microvesicular		5/7 [33] 1/1 [36]	2/2 [43] 22/48 [59]	
	Not further specified		1/4 [20] 11/11 [21] 1/9 [23] 7/17 [24] 1/10 [25]	2/12 [27] 1/1 [55] 9/14 [28] 6/10 [35]	
Congestion			1/4 [20] 8/11 [21] 4/9 [23] 2/12 [27] 1/1 [55]	11/14 [28] 4/80 [29] 7/7 [33] 10/10 [35] 1/1 [41]	Right-sided cardiac failure (may be secondary to effects of COVID-19 or may represent evidence of pre-existing cardiorespiratory disease)
Hepatocyte injury	Cellular death	Not further specified	0/4 [20]	1/3 [56]	Various causes
		Alcohol/ non-alcohol related steatohepatitis	3/17 [24]		
		Massive	1/11 [21]		Seen in severe hepatic injury due to a variety of causes
		Patchy/ focal	2/11 [21] 7/7 [33]	¼ [34] 2/2 [43]	May be seen in acute viral hepatitis (“spotty necrosis”; usually associated with lymphocytes)
		Periportal & centrilobular	1/4 [34]		Periportal necrosis may be due to interface hepatitis. Centrilobular necrosis is attributable to hypoperfusion injury but may also be seen in drug and toxin-mediated injury
		Centrilobular	4/11 [21] 5/17 [24] 4/14 [28]	3/10 [35] 1/1 [36]	
	Mild ballooning degeneration		4/7 [33]		May be seen in cholestasis or marked hepatitis
	Kupffer cell activation		2/4 [20] 10/10 [21] 1/4 [34]	2/2 [43] 5/10 [35]	Non-specific finding secondary to hepatocyte injury
	Syncytial hepatocytes		2/2 [43]		Non-specific finding secondary to hepatocyte regeneration
Inflammation	Not further specified		0/4 [20]		No evidence of hepatitis
	Lymphoplasmacytic, not further specified		1/1 [22]		Seen in a variety of pathologies including resolving acute hepatitis, autoimmune hepatitis, hepatitis B/C, biliary disease
	Parenchymal	Lobular lymphocytic	2/4 s [34] 24/48 [59]		May be seen in acute viral hepatitis (CMV, EBV), autoimmune hepatitis, and primary biliary cholangitis
		Lobular lymphocytic & neutrophilic	1/2 [43]		Neutrophilic inflammation may be seen in steatohepatitis (especially alcohol-related)
		Lobular neutrophilic	1/14 [28]		
		Sinusoidal neutrophilic	6/10 [35]		
		Neutrophil microabscess	1/1 [41]		Seen in human cytomegalovirus (CMV) infection but may also be seen in other settings, such as ascending cholangitis and seeding from a septic source
		Not specified	0/1 [36]		
	Periportal	Lymphocytic	8/11 [21] 4/14 [28] 1/4(C/SLL) [34]	1/2 [43] 32/48 [59]	May be seen in a wide variety of hepatitises
		Lymphoplasmacytic	NS/10 (“minimal”) [25]		
		Not specified	7/7 [33] 0/1 [36]	9/10 [35]	
	Vascular	Lymphocytic endothelialitis	1/3 [56]		
Sinusoidal abnormalities	Dilatation		7/7 [33]	2/4 [34]	Seen in hypercoagulability syndromes and venous flow abnormalities
	Intrasinusodal fibrin thrombi		1/10 [35] 0/2 [43]	13/48 [59]	Non-specific finding implicated in the pathogenesis of hepatic congestion
Ductal/canalicular abnormalities	Cholestasis		8/11 [21] 2/7 [33]	0/2 [43]	Non-specific finding that may be seen in sepsis amongst other aetiologies
	Ductular reaction with lymphocytic inflammation		7/11 [21]		Non-specific finding secondary to acute biliary obstruction
	Ductular reaction without inflammation		1/11 [21]		
Haemophagocytosis			1/4 [20]	0/4 [50]	Non-specific finding that may be seen in sepsis and other systemic haemophagocytic disorders
Thrombosis			1/11 [21] 35/48 [59]		Implies a pro-coagulant state (systemic/ localised)
Macroscopic impression of liver shock			3/12 [27]	NS/80 [29]	Secondary to hypoperfusion
Hepatomegaly (aetiology not specified)			1/1 (“minimal”) [22]	1/4 [27] 0/4 [50]	Significance uncertain

**Table 4:** Prevalence of steatosis, venous congestion and cirrhosis in pre-pandemic and COVID-19 post-mortem cohorts. Compared with pre-pandemic PM cohorts, our COVID-19 PM cohort had a significantly higher frequency of steatosis [X^2^(1, *n* = 2086) =7.72 *p* < 0.005] and cirrhosis [X^2^(1, *n* = 1862) = 24.22 *p* < 0.001], but not venous congestion [X^2^(1, *n* = 360) = 1.55 *p* > 0.05]. When aggregating our study with those of other published COVID PMs there was a significantly higher frequency of steatosis [X^2^(1, *n* = 2250) =22.24 *p* < 0.00001], cirrhosis [X^2^(1, *n* = 1959) =27.55 *p* < 0.00001] and venous congestion [X^2^(1, *n* = 520) = 21.07 *p* < 0.00001] compared to pre-pandemic PM cohorts. Considering COVID-19 published studies only, compared with pre-pandemic PM cohorts, there was a significantly higher frequency of steatosis [X^2^(1, *n* = 2231) =16.83 *p* < 0.0001], cirrhosis [X^2^(1, *n* = 1940) = 14.88 *p* < 0.001] and venous congestion [X^2^(1, *n* = 501) =21.49 *p* < 0.00001].

PM Cohort	Steatosis	Venous Congestion	Cirrhosis
COVID-19 PM cases in our study (Table 1)	12/19 (63%) *p* < 0.05	5/19 (26%) *p* > 0.05	4/19 (21%) *p* < 0.00001
COVID-19 PM cases in published studies (Table 3)	80/164 (49%) *p* < 0.0001	54/160 (34%) *p* < 0.00001	9/97 (9.3%) *p* < 0.001
COVID-19 PM cases in our study and published studies	92/183 (50%) *p* < 0.00001	59/179 (33%) *p* < 0.00001	13/116 (11%) *p* < 0.00001
Aggregate data derived from all pre-pandemic PM studies detailed in the rows below	682/2067 (33%)	53/341 (16%)	47/1843 (2.6%)
Unnatural causes of death, including sudden death and car accidents [70]	156/498 31.%	Not measured	6/498 1.2%
Non burn trauma [76]	108/224 48%	Not measured	
Multiple causes: trauma (35%), acute myocardial infarction (30%), opiate overdose (13%), cerebrovascular accidents (4%), infectious diseases (3%) and others (15%) [71]	283/896 31.6%	Not measured	7/896 (0.8%)
Causes of death listed as “diverse”. Paper specifies that liver disease was not primary cause of death in any included case. NB this was a study of dissection room cadavers, not autopsies [72]	24/68 35%	Not measured	3/68 4.4%
Cardiovascular diseases 68%, respiratory illnesses 25%, gastrointestinal disorders 5% and cerebrovascular disease 2% [76]	6/40 15%	Not measured	3/40 7.5%
Road/railway accidents, burns, drowning, hanging and poisoning [71]	24/70 34%	19/70 27%	8/70 11%
Road accidents, poisoning [73]	25/100 25%	12/100 12%	4/100 4%
Medicolegal autopsies [74]	46/121 38%	9/121 7.4%	8/121 6.6%
Road traffic Accidents (*n* = 35), poisoning (*n* = 5), hanging (*n* = 3), suspicious death (*n* = 1), Myocardial infarction (*n* = 2), drowning (*n* = 2), burns (*n* = 1) and on railway (*n* = 1) [75]	10/50 20%	13/50 26%	8/50 16%

**Table 5:** Results of analysis of two post-mortem liver function tests, albumin and alanine aminotransferase (ALT), as a function of length of hospital admission and patient survival data. Statistically significant results below the 0.05 level are underlined.

Length of Patient Admission (days)	Abnormal Albumin on Admission	*p* Value (Chi Squared) for Significance of Abnormal vs. Normal Albumin Compared with Poorer Patient Outcomes	Albumin Level (g/L) Mean ± SD [Normal Range: 35–50 g/L]	Abnormal Alanine Transaminase (ALT) on Admission	*p* Value (Chi Squared) for Significance of Abnormal vs. Normal ALT Compared with Poorer Patient Outcomes	Alanine Transaminase (iU/L) Mean ± SD [Normal Range: 10–49 iU/L]
**<7**	38/54 (70.4%)	0.00043	29 ± 3.9	16/54 (26.6%)	0.42	76.8 ± 45.3
**7–13**	46/52 (88.5%)	0.020	27.8 ± 4.1	13/52 (25.0%)	0.55	93.7 ± 59.8
**14–20**	37/43 (86.0%)	0.028	26.8 ± 3.7	14/43 (32.6%)	0.15	98.5 ± 133.6
**>21**	35/39 (89.7%)	0.081 *(All survivors vs. deceased)*	24.5 ± 5.8	13/39 (33.3%)	0.0135 *(All survivors vs. deceased)*	66.2 ± 29.2
**Deceased**	80/88 (90.9%)		24.6 ± 5.8	14/88 (15.9%)		101.1 ± 88.7
**Total**	236/276 (85.5%)		26.2 ± 5.2	70/276 (25.3%)		87.3 ± 78.6
**One-way ANOVA *p* value**			0.000012			0.73

## Data Availability

Full data are presented in this study.
